# A novel homozygous mutation in the *PADI6* gene causes early embryo arrest

**DOI:** 10.1186/s12978-022-01495-7

**Published:** 2022-09-10

**Authors:** Xiaoxia Wang, Huimin Zhu, Yi He, Jun Zeng, Jing Zhao, Qiuping Xia, Lingqian Wu, Zhongyuan Yao, Yanping Li

**Affiliations:** 1 Center for Medical Genetics & Hunan Key Laboratory of Medical Genetics, School of Life Sciences, Central South University, Changsha, 410078 Hunan China; 2 Department of Reproductive Medicine, Xiangya Hospital, Central South University, Changsha, 410000 Hunan China; 3 Clinical Research Center for Women’s Reproductive Health in Hunan Province, Changsha, 410000 Hunan China; 4 Department of Neurosurgery, Xiangya Hospital, Central South University, Changsha, 410008 Hunan China; 5 The Fourth Affiliated Hospital, Zhejiang University School of Medicine, Yiwu, 322000 Zhejiang China

**Keywords:** Infertility, Early embryo arrest, Peptidylarginine deiminase type VI, Mutation, Subcortical maternal complex

## Abstract

**Background:**

It has been proved that mutations in the *PADI6* gene can cause early embryo arrest. This study describes a newly discovered mutation in *PADI6* that expands the genetic spectrum of early embryo arrest.

**Methods:**

Peripheral blood of a patient diagnosed with early embryo arrest was collected for whole-exome sequencing. Sanger sequencing was performed to confirm this mutation. The effects of the variant were investigated in human embryonic kidney 293T (HEK293T) cells using western blotting, real-time quantitative polymerase chain reaction, and immunofluorescence.

**Results:**

A novel homozygous mutation in *PADI6* was identified in the proband. The patient carried a frameshift insertion mutation c.558dupA (p.Thr187Asnfs*48), which was located in the protein arginine deiminase middle domain. The variant destroyed PADI6 protein expression and reduced *PADI6* mRNA expression in HEK293T cells.

**Conclusions:**

The newly identified mutation in *PADI6* accounts for early embryo arrest. It expands the spectrum of genetic causes and phenotypes of infertility in humans. These findings also provide an additional possible diagnostic marker for patients with recurrent in vitro fertilization/intracytoplasmic sperm injection failure.

**Supplementary Information:**

The online version contains supplementary material available at 10.1186/s12978-022-01495-7.

## Plain Language Summary

Some infertile patients experience multiple in vitro fertilization/intracytoplasmic sperm injection (IVF/ICSI) failure owing to recurrent early embryo arrest. However, the underlying mechanisms remain largely unknown. Due to the development of whole-exome sequencing, early embryo arrest has been confirmed as a type of Mendelian disease. This study aimed to identify the genetic cause of early embryo arrest in patients and to expand the genetic spectrum. Furthermore, it can help doctors offer better suggestions to such patients and prevent patients from suffering from multiple IVF/ICSI failures.

**Supplementary Information:**

The online version contains supplementary material available at 10.1186/s12978-022-01495-7.

## Background

Infertility affects 70 million couples of reproductive age in the world [1]. The only way they can produce their own babies is through assisted reproductive technology. To date, approximately 8 million babies have been produced by in vitro fertilization/intracytoplasmic sperm injection (IVF/ICSI) [2]. However, some couples with infertility experience multiple IVF/ICSI failures for unknown reasons. Successful establishment of human pregnancy requires that mature oocytes to fuse with sperms. Not all zygotes have the opportunity to develop viable embryos. It is estimated that only about 40–70% of human embryos develop into viable embryos [3]. This phenomenon is early embryo arrest referred to as preimplantation embryo lethality, and arrested cleaved embryos cannot form blastocysts [4]. Unexplained early embryonic arrest is the major cause.

Previously, genes related to early embryo arrest were only investigated in animal models. The cause in humans remains largely elusive due to ethical concerns. Contributing to the development of IVF/ICSI, investigators can observe human oocytes/embryos in vitro. Furthermore, several genes, including PAT1 homolog 2 (*PATL2*), tubulin beta 8 class VIII (*TUBB8*), TLE family member 6 (*TLE6*), cell division cycle 20 (*CDC20*), peptidyl arginine deiminase 6 (*PADI6*), NLR family pyrin domain containing 2 (*NLRP2*), NLR family pyrin domain containing 5 (*NLRP5*), KH domain containing 3 like (*KHDC3L*), REC114 meiotic recombination protein (*REC114*) and meiotic double-stranded break formation protein 1 (*MEI1*) [4–6], have been proved to cause early embryo arrest in human and then female infertility.

Maternal effect factors are essential for embryogenesis. Early human embryos (before the eight-cell stages) are transcriptionally silent and utilize maternally provided transcripts and proteins during the maturation process of growing oocytes [7]. Human subcortical maternal complex (SCMC) is a multiprotein complex uniquely located in the subcortex of the mammalian oocytes and embryos, which mainly consists of eight proteins, including *TLE6*, *NLRP2*, *NLRP5*, *NLRP7*, oocyte permitting embryonic development (*OOEP*), *KHDC3L* and *PADI6*, and zinc finger BED-type containing 3 (*ZBED3*) [8, 9]. SCMC is an important maternal-effect factor. They are highly expressed in the oocytes and early embryos, and usually begin to degrade due to lack of transcriptional compensation after the embryonic genome becomes active [10], playing an important role in orchestrating oocyte-embryo transition.

A series of studies have shown that SCMC genes are involved in reproductive failure, such as early embryonic arrest, fertilization failure, and recurrent hydatidiform moles [11–13]. However, this can only account for a small number of patients. Therefore, novel mutations need to be investigated to establish the role of SCMC in embryogenesis and lay the foundation for the genetic diagnosis of female infertility.

In the present study, a novel mutation in the SCMC gene *PADI6* was identified using whole-exome sequencing (WES) in a patient with multiple IVF/ICSI failures. This study broadened our understanding of the genetic and phenotypic characteristics of early embryonic arrest.

## Methods

### Human subjects

One patient diagnosed with early embryo arrest during common IVF treatment was recruited from the Department of Reproductive Medicine, Xiangya Hospital, Central South University. Participants with normal embryo development in IVF/ICSI cycles were also recruited as the control group. The study was approved by the Institutional Review Board of the Department of Reproductive Medicine, Xiangya Hospital (No.2021005). Informed consent was obtained from all the recruited participants.

### DNA extraction

Venous blood samples of patients and 100 controls were collected in EDTA blood tubes. Genomic DNA was extracted according to the standard protocol of the QIAamp DNA Blood Midi Kit (QIAGEN). The DNA concentration and purity were measured using a NanoDrop spectrophotometer (Thermo Fisher Scientific).

### WES

The Exome was captured using xGen Exome Research Panel v2 (22307, Integrated DNA Technologies, San Diego, California, USA) and sequencing was performed with the Novaseq system (PE150, Illumina, San Diego, California, USA). Sequences were aligned to a human reference sequence (human genome 19, hg19) using Bowtie2. After filtering the unmapped variants, single nucleotide polymorphism (SNP) and insertion and deletion (InDel) polymorphisms were detected using FreeBayes. The variants were annotated using the ANNOVAR software. The frequency of corresponding mutations was determined using the 1000 Genomes Project (1000G), Exome Aggregation Consortium (ExAC) and Exome Aggregation Consortium_East Asian (ExAC_EAS) databases, and the functional effects of the mutations were predicted using the in silico algorithms MutationTaster. The filtering criteria were as followed: (1) variants with an allele frequency lower than 0.1% in the 1000G and ExAC databases; (2) exonic nonsynonymous, splicing, or coding indels; and (3) variants with a previously reported embryogenesis-related function. The American College of Medical Genetics and Genomics (ACMG) was used to assess the mutations. HomozygosityMapper (http://www.homozygositymapper.org/) was used to determine the existence of candidate homozygous variants in the proband [14].

### Sanger sequencing

The targeted variant in *PADI6* was amplified by polymerase chain reaction (PCR) using the ProFlex™ Base (Applied Biosystems, Singapore). Specific primers flanking the mutation sites were listed in Additional file 1: Table S1. The mutation variant was then confirmed by Sanger sequencing in the patients and controls.

### Evolutionary conservation analysis and molecular modelling

Evolutionary conservation was assessed using Clustal Omega software (https://www.ebi.ac.uk/Tools/msa/clustalo/). DNA structures was drawn using the Gene Structure Display Server (http://gsds.gao-lab.org/) [15]. The Pfam database (http://pfam.xfam.org/) was used to analyze the protein domains. In addition, wild-type (wt) *PADI6* was built using SWISS-MODEL software (https://swissmodel.expasy.org/) in the automated model and mutated *PADI6* was mapped using SWISS-Pdb Viewer.

### Plasmid construction and mutagenesis

Plasmids were synthesized by Sangon Biotech Co., Ltd (Shanghai, China). Briefly, the full-length coding sequences of wild-type and mutant *PADI6* (NM_207421.4) were synthesized and inserted into the p.cDNA3.1(+) vector with a C-terminal Flag tag. Sanger sequencing confirmed the wild-type and mutant clones.

### Cell culture and transfection

Human embryonic kidney 293T (HEK293T) cells were kindly provided by the Center for Medical Genetics & Hunan Key Laboratory of Medical Genetics, School of Life Sciences, Central South University. Cells were cultured in Dulbecco’s modified Eagle’s medium (DMEM, Biological Industries) supplemented with 10% fetal bovine serum (Biological Industries) and 1% penicillin/streptomycin (Gibco) in a humidified incubator with a 5% CO_2_ atmosphere at 37 °C. Transient transfections were performed using the Lipofectamine 2000 reagent (Invitrogen), according to the manufacturer’s instructions. DNase and RNase water of equal volumes were added into the blank groups as controls.

### Western blotting

The cells were washed thrice with cold PBS after transfection for 48 h. Cells were harvested and lysed with RIPA buffer (Beyotime Biotechnology) containing with 1× protease inhibitor cocktail (Beyotime Biotechnology). After incubating on the ice for 20 min and centrifuging at 12,000 rpm at 4 °C for 20 min, protein lysates were collected in a new centrifuge tube. After quantification using a bicinchoninic acid kit (Beyotime Biotechnology), equal amounts of samples were denatured in protein loading buffer (NCM Biotech) and heated at 100 °C for 10 min. Protein samples were separated by 9% sodium dodecyl sulfate-polyacrylamide gel electrophoresis (SDS-PAGE) and transferred onto PVDF membranes (Millipore). The membranes were blocked in 5% skim milk diluted in 1× Tris-buffered saline with Tween 20 (TBST) for 1 h and incubated at 4 °C overnight with primary antibodies against Flag (1:1000, 8146S, Cell Signaling Technology) or GAPDH (1:10,000, 60004-1-Ig, Proteintech). After 1 h incubation with the corresponding secondary antibodies (1:10,000, 7076S, Cell Signaling Technology) for 1 h at room temperature on the secondary day, the membranes were visualized using Amersham ImageQuant 800 (Cytiva, United States) after detection with BeyoECL Plus (Beyotime).

### Immunofluorescence

After transfection for 48 h, cells on slides were fixed with 4% paraformaldehyde for 20 min, and then incubated in permeabilizing solution (0.02% Triton X-100 in PBS) for 15 min and blocking buffer (5% goat serum in PBS) for 1 h. Anti-Flag antibody (1:400, 8146S, Cell Signaling Technology) was added at 4 °C overnight. On the secondary day, the cells were incubated with CoraLite488-conjugated goat anti-mouse IgG(H+L) (1:400, SA00013-1, Proteintech) for 1 h to visualize PADI6-Flag staining. DAPI Fluoromount-G (SouthernBiotech) was used to label DNA. Finally, the cells were imaged on a fluorescence microscope (Leica, Germany).

### RNA isolation and real-time quantitative polymerase chain reaction (RT-qPCR)

Total RNA was extracted from HEK293T cells 48 h after transfection using the Total RNA Purification Kit (SimGen). Reverse transcription and amplification were performed using the Evo M-MLV RT Mix Kit (Accurate Biotechnology), and RT-qPCR was performed using the SYBR Green Premix Pro Taq HS qPCR Kit (Accurate Biotechnology) according to the manufacturer’s instructions. All reactions were performed with at least three technical replicates. The relative expression level was calculated using 2^−△△Ct^ normalized to endogenous GAPDH expression. Primers used in the experiments are listed in Additional file 1: Table S1.

### Statistical analysis

All experiments were independently conducted in triplicate. RT-qPCR analysis was performed using Student’s t-tests when comparing experimental groups. Statistical analyses were performed using GraphPad Prism 8.0. *P* < 0.05 was considered as statistically significant.

## Results

### Clinical characteristics of the affected individual

A novel homozygous *PADI6* mutation was identified in an infertile patient with recurrent IVF/ICSI failure. The proband had been diagnosed with primary infertility for several years, and had normal menstruation and hormone levels (Additional file 1: Table S2). The family pedigree is shown in Fig. 2a.

**Fig. 1:**
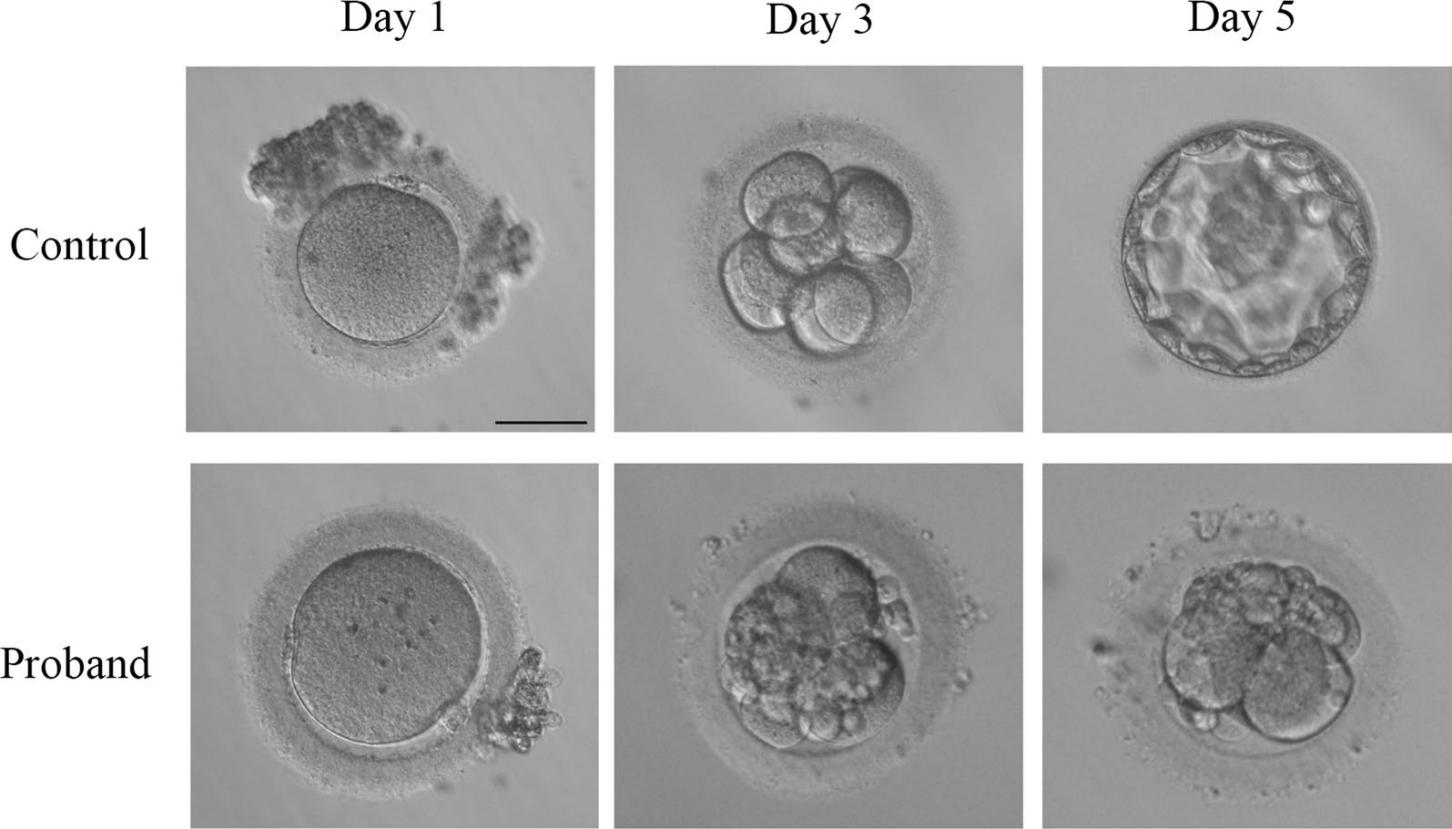
Morphologic characteristics of oocytes and embryos from the affected individual and normal infertile patient. Scale bar = 50μm

The proband was 34 years old with a 15-year infertile history at examination. Initially, it was found that the two sides of the fallopian tubes were not completely obstructed. She had tried four cycles of ovulation monitoring, but had been not pregnant. The couple then accepted an ICSI attempt at another hospital, but it failed because of the lack of available embryos. At our hospital, an antagonist protocol with ICSI treatment was administered. Fifteen oocytes were retrieved, including three germinal vesicles (GV) and 12 MII oocytes. A total of 11 oocytes were fertilized and ten of them formed cleaved embryos, although all of them were of poor quality (Table 1). Two 3cells/II embryos were transferred on the Day 2. In the end, no viable blastocysts were obtained in prolonged cultivation, and no pregnancy was established, indicating early embryonic arrest (Table 1, Fig. 1).

**Table 1:** Oocyte and embryo characteristics of the patient

Patient	Age (years)	Infertility duration (Years)	Retrieved oocytes	GV oocytes	MI oocytes	MII oocytes	Embryo outcomes on Day 1	Embryo outcomes on Day 2	Embryo outcomes on Day 3	Embryo outcomes on Day 5
Proband	34	15	15	3	0	12	8 × 2PN, 1 × 1PN, 1 × 3PN, 1 × 2-cell	1 × 2-cell, 2 × 3-cell (being implanted), 1 × 4-cell, 3 × fragmentation, 1 × polynucleation	2 × 3-cell (developed from MII oocyte and an embryo with polynuleation, respectively), 1 × 4-cell, 1 × 5-cell	No viable blastocytes

GV, germinal vesicle; MI, metaphase I; MII, metaphase II; PN, pronucleus

### Identification of novel mutations in PADI6

**Fig. 2:**
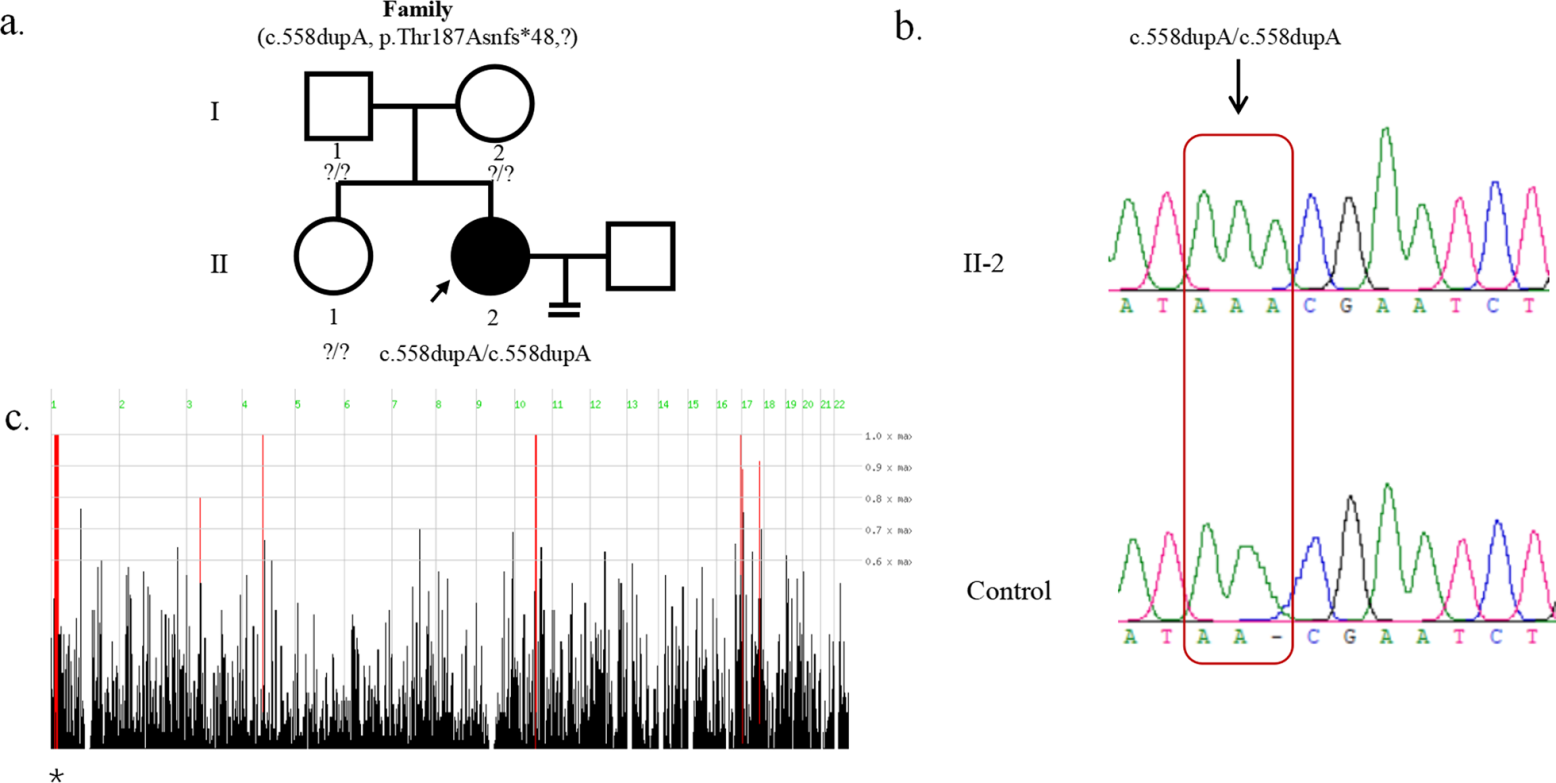
Identification of *PADI6* mutation in one affected family. **a** Pedigree of a family who carry the PADI6 mutation. The affected individual shows homozygous mutation. Squares indicate male individuals, and circles indicate female individuals. The black circle with an arrow indicates the affected individual, and blank symbols indicate unaffected individuals. The equal sign represents infertility. **b** Sanger sequencing confirmation of the proband and control. **c** Homozygosity mapping of the proband. Homozygous regions harboring the strongest signal are indicated in red, and the asterisk (*) denotes where *PADI6* is located

In the proband, a homozygous frameshift insertion mutation was identified in exon 6 of *PADI6* (NM_207421.4: c.558dupA: p.Thr187Asnfs*48) (Fig. 2a). Sanger sequencing was performed to confirm that the mutation was homozygous (Fig. 2b). Homozygosity mapping detected a strong signal in patient (II-2) in the region of chromosome 1 that contained *PADI6* (Fig. 2c). Because the DNA samples of their families were unavailable, the mutation inheritance was unknown.

### Conservative and in silico analysis of the variant effects on protein function

**Fig. 3:**
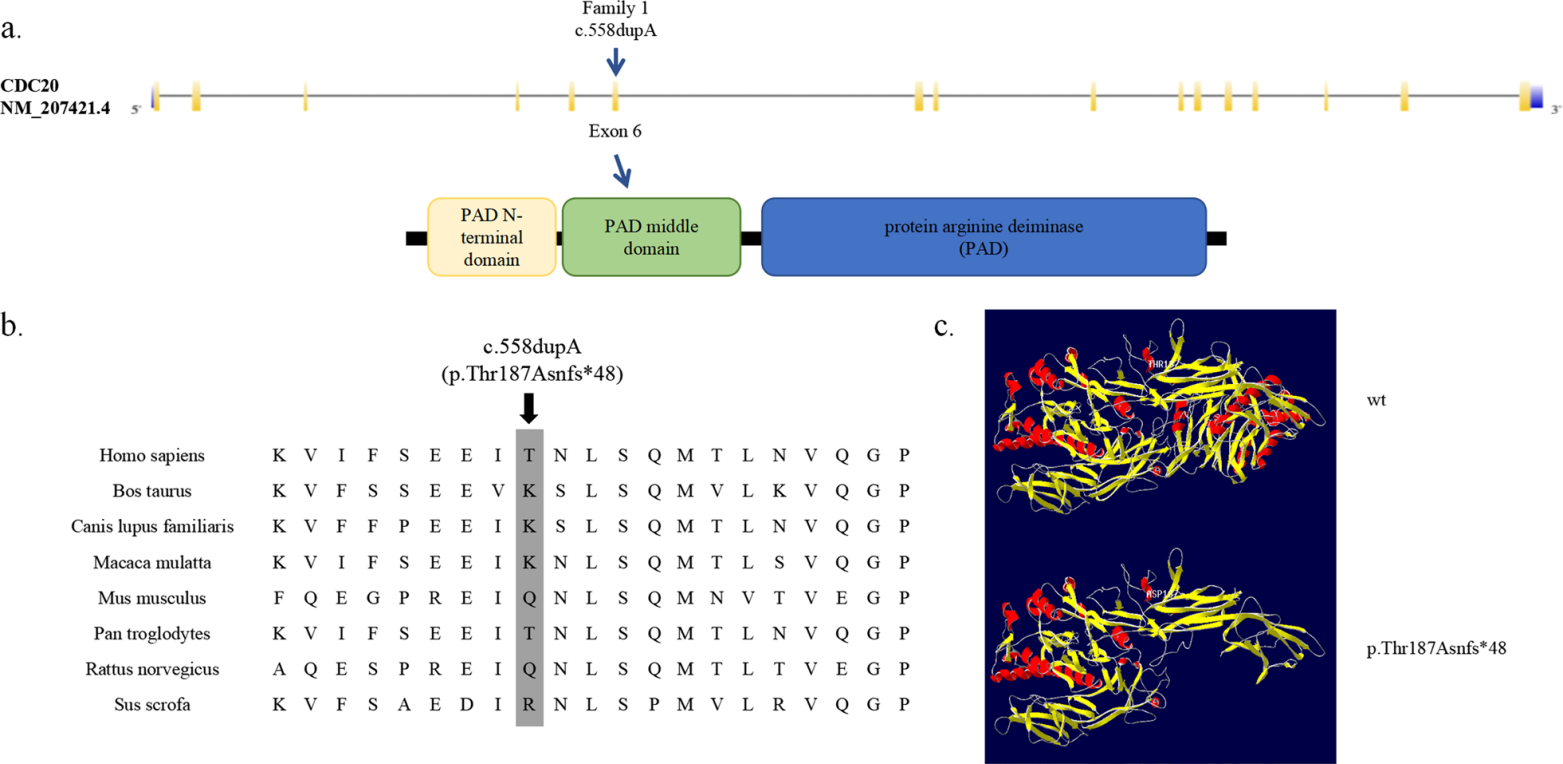
The locations and conservation of mutated residues position in PADI6. **a** The localization of the mutations is indicated in the genomic and protein structure of PADI6. **b** Conservation of amino acids in PADI6 among different 8 mammalian orthologs. **c** Protein conformation prediction of the effect of mutation in PADI6. The view shows the structure comparison of structure of the frameshift insertion mutation in PADI6 protein with wild-type PADI6 protein

The mutation site was located in exon 6 of the *PADI6* gene (Fig. 3a). The amino acid at position p.Thr187 of *PADI6* was not conserved among the eight mammalian orthologs (Fig. 3b). The mutation was absent in the 1000G, ExAC, and ExAC_EAS databases (Table 2). The variant that we identified was not predicted by MutationTaster (Table 2). According to the three-dimensional (3D) structure of *PADI6*, the frameshift insertion mutation caused the replacement of threonine with asparagine at position 187, resulting in a truncated protein (Fig. 3c). The mutation site was in the α-helix, which might influence the formation and stability of the α-helix. The mutation was pathogenic according to the ACMG guidelines.

**Table 2:** Overview of the *PADI6* mutations identified in the affected individual

Patient	Genomic DNA position	Exon	cDNA change	Protein change	Exonic function	1000G	ExAC	ExAC_EAS	MutationTaster	ACMG classification
Proband	Chr.1:17708465	6	c.558dupA	p.Thr187Asnfs*48	Frameshift insertion	NA	N/A	N/A	N/A	Pathogenic (PVS1, PM2, PM4)

1000G, 1000 Genomes Project; ExAC, Exome Aggregation Consortium; EAS, East Asian; ACMG, American College of Medical Genetics and Genomics

### Effects of the variant on protein and mRNA expression in HEK293T cells

**Fig. 4:**
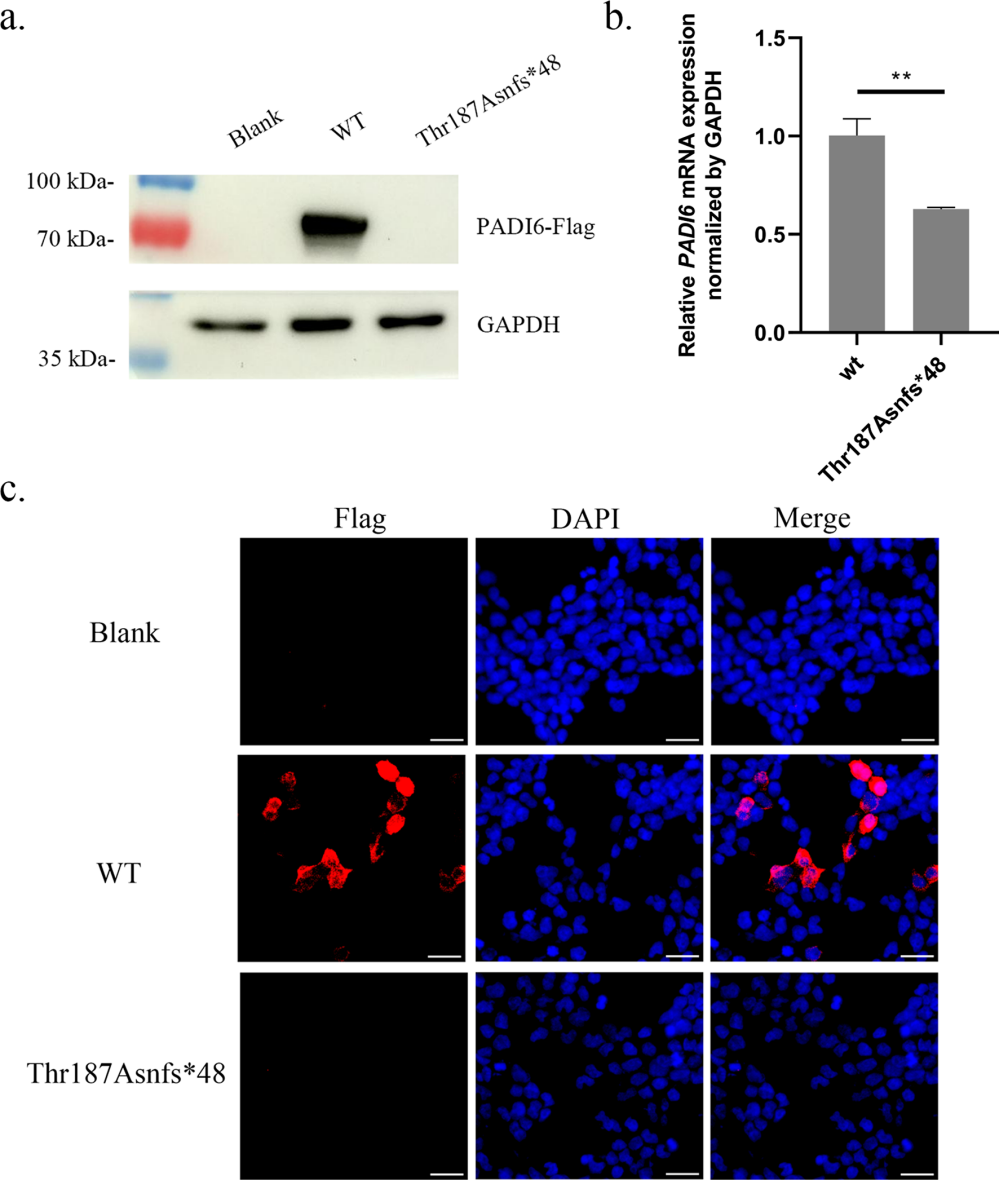
Effects of the variant on PADI6 protein and mRNA expression in vitro. **a** Western blot of PADI6 protein expression in HEK-293T cells transfected with Flag-tagged wild-type and mutant plasmids. GAPDH was used as the internal control. **b** RT-qPCR analysis of wild-type and mutant *PADI6* mRNA level. The bars showed the mean of three separate measurements, and the error denoted the SEM. **c** Immunofluorescence of wild-type and mutant PADI6. The experiment was performed with three independent biological replicates yielding similar results. **P < 0.01

To evaluate the functional influences of the PADI variant in vitro, PADI6 wild-type and mutant plasmids identified in this study were constructed and transiently transfected into HEK293T cells. Western blotting and immunofluorescence analysis demonstrated that compared with the wild-type group, the p.Thr197Asnfs*48 protein could not be expressed (Fig. 4a and c). RT-qPCR showed a significant reduction in the mRNA levels of the p.Thr197Asnfs*48 variant (Fig. 4b).

## Discussion

In the present study, a novel homozygous mutation (NM_207421.4: c.558dupA: p.Thr187Asnfs*48) in gene *PADI6* was discovered in a patient diagnosed with primary infertility due to early embryonic arrest. We constructed wild-type and mutant *PADI6* plasmids and transfected them into HEK293T cells to investigate the effects of the variant on protein and mRNA expression in vitro. The results showed that p.Thr187Asnfs*48 could cause a significant reduction in mRNA levels and did not induce protein expression. This discovery expanded the genetic spectrum of early embryonic arrest diagnosis.

The PADI family is comprised of five members, including *PADI1*, *PADI2*, *PADI3*, *PADI4*, and *PADI6*. Among these, *PADI6* is exclusively expressed in oocytes and early embryos [9]. *PADI6* is one of SCMC components that localizes to other components in oocytes and early embryos. It encodes a peptidylalarginine deiminase. Peptidylarginine deiminase belongs to a class of Ca^2+^-dependent enzyme that convert arginine residues to citrulline, which is called citrullination and is crucial for the formation of rigid structures such as hair, skin, and myelin sheath [16, 17]. It is highly conserved in their amino acid sequence of mammalian peptidylarginine deiminases [18]. *PADI6* knockout mouse studies confirm that *PADI6* null oocytes lack a specialized cytoskeletal structure called as cytoplasmic lattices and consequently have abnormal organelle positioning and redistribution [19]. Furthermore, the stored *PADI6* mRNAs are largely degraded in the zygote [20, 21], altering spindle microtubule assembly and movement to mediate proper cleavage in early embryo development [20, 22]. Therefore, *PADI6* null embryos were arrested between the two-cell and four-cell stage [20].

In 2016, Xu et al. first reported that *PADI6* variants were associated with embryonic arrest due to embryonic genome activation failure in humans [23]. To date, studies have reported six homozygous mutations or four compound-heterozygous mutations that caused early embryo arrest (Additional file 1: Table S3). Other studies confirm that *PADI6* does not only cause (early) pregnancy losses and recurrent hydatidiform moles, but maternal effect variants in this gene cause chromosomal aberrations and disturbed imprinting, producing children with Beckwith–Wiedemann syndrome or Silver–Russell syndrome (Additional file 1: Table S3). These studies indicated that different mutations in *PADI6* cause phenotypic variability in patients.

In this study, a homozygous mutation c.558dupA (p.T187Nfs*48) caused early embryonic arrest in the proband (II-2), resulting in 3- or 5-cell arrested embryos on Day 3 and no viable blastocytes on Day 5, which was congruent with previous reports [8, 9, 23–25]. Due to the limitation of the PADI6 antibody for immunofluorescence, we did not investigate the in vivo expression of PADI6 in the oocytes of the patient. However, bioinformatics predicted that the mutation could result in a truncated protein because of a premature stop codon. Although most reported mutations are localized in the protein arginine deiminase (PAD) domain, this novel homozygous mutation is in the PAD middle domain. In addition, the variant displayed a significant reduction in mRNA levels and no protein expression in vitro experiments. It was possibly caused by nonsense-mediated mRNA and protein decay. Therefore, we speculate that the variant could result in no expression of PADI6 in the patient’s oocytes, finally leading to unstable SCMC and embryo developmental arrest.

For patients diagnosed with infertility caused by PADI6 mutations, genetic counselling is recommended. Oocyte donation may be a feasible option for preventing an adverse pregnancy and unnecessary IVF treatment. Several studies have shown that complementary RNAs (cRNAs) injection can rescue abnormal oocyte development, resulting in blastocytes with normal copy number variations [26, 27]. Although no rescue study has been performed to treat oocyte/embryo with PADI6 mutations, it may be a promising gene therapy for these patients.

## Conclusions

A novel homozygous mutation in PADI6 was identified in the present study, which can account for early embryo arrest caused by genetic abnormalities and expand the spectrum of genetic causes and phenotypes of human infertility. These findings also provide an additional possible diagnostic marker for patients with recurrent IVF/ICSI failure.

## Supplementary Information

**Additional file 1: Table S1.** *PADI6* primer sequences. **Table S2.** Sex hormone characteristics of the proband during the controlled ovarian stimulation. **Table S3.** Overview of mutation sites and associated phenotypes of *PADI6* in previous studies.

## Data Availability

The datasets generated during and/or analyzed during the current study are available from the corresponding author on reasonable request.

## Abbreviations

HEK: Human embryonic kidney
IVF: In vitro fertilization
ICSI: Intracytoplasmic sperm injection
*PATL1*: *PAT1* Homolog2
*TUBB8*: Tubulin beta 8 class VIII
*TLE6*: *TLE* Family member
*CDC20*: Cell division cycle 20
*PADI6*: Peptidyl arginine deiminase 6
*NLRP2*: *NLR* Family pyrin domain containing 2
*NLRP5*: *NLR* Family pyrin domain containing 5
*KHDC3L*: *KH* Domain containing 3 like
*REC114*: *REC* Meiotic recombination protein
*MEI1*: Meiotic double-stranded break formation protein 1
SCMC: Subcortical maternal complex
*OOEP*: Oocyte permitting embryonic development
*ZBED3*: Zinc finger BED-type containing 3
WES: Whole-exome sequencing
hg19: Human genome 19
SNP: Single nucleotide polymorphism
InDel: Insertion and deletion
1000G: 1000 Genomes Project
ExAC: Exome Aggregation Consortium
ExAC_EAS: Exome Aggregation Consortium_East Asian
ACMG: American College of Medical Genetics and Genomics
PCR: Polymerase chain reaction
GV: Germinal vesicles
3D: Three dimension
PAD: Protein arginine deiminase
cRNAs: Complementary RNAs
