# Supplementary material for: A novel homozygous mutation in the PADI6 gene causes early embryo arrest
Source: Reprod Health. 2022 Sep 10;19:190. doi: 10.1186/s12978-022-01495-7 (PMC9463787; doi:10.1186/s12978-022-01495-7)
Supplement: Supplementary file 1 — Additional file 1: Table S1. PADI6 primer sequences. Table S2. Sex hormone characteristics of the proband during the controlled ovarian stimulation. Table S3. Overview of mutation sites and associated phenotypes of PADI6 in previous studies. [file 12978_2022_1495_MOESM1_ESM.docx]

Table S1. *PADI6* primer sequences

| Gene name | sequence |
| --- | --- |
| *PADI6*-exon6-F | TCGAGGTAGGCCCTGTTCTG |
| *PADI6*-exon6-R | GAGCTGGCACAAGAACACTCA |
| *PADI6*-RT-F | ATGCCGTTTGTGTGTTGGG |
| *PADI6*-RT-R | TCTCAGAAATCACCGTGTTGG |
| *GAPDH*-RT-F | CAAATTCCATGGCACCGTCA |
| *GAPDH*-RT-R | CAAATTCCATGGCACCGTCA |

*PADI6*, peptidylarginine deiminase type VI; *PADI6* (NM_207421.4) was used for primer design.

Table S2. Sex hormone characteristics of the proband during the controlled ovarian stimulation

| Patient | Day | FSH (IU/ml) | LH (IU/ml） | E_2_ (pg/ml) | P (ng/ml) |
| --- | --- | --- | --- | --- | --- |
| Proband | MC3 | 4.9 | 4.9 | 29.3 | 0.1 |
|  | hCG triggering | NA | 1.5 | 3002 | 0.7 |

MC3, menstrual cycle day 3; FSH, follicle stimulating hormone; LH, luteinizing hormone; E_2_, estrogen; P, progesterone; NA, not available.

Supplemental Table 3. Overview of mutation sites and associated phenotypes of *PADI6* in previous studies

| Phenotype | Hom/com-het | cDNA change | Protein change | Study |
| --- | --- | --- | --- | --- |
| Early embryo arrest | Hom | c.831_832del | p.Ser278Profs*59 | (Liu et al., 2021); (Zheng et al., 2021) |
|  | Com-het | c.1117A>C | p.Thr373Pro | (Zheng et al., 2020) |
|  |  | c.1708C>T | p.Arg570Cys |  |
|  | Hom | c.1521dupC | p.Ser508Qfs*5 |  |
|  | Com-het | c.866C>T | p.Pro289Leu | (Wang et al., 2018) |
|  |  | c.1895C>T | p.Pro632Leu |  |
|  | Hom | c.1124dupT | p.Leu375Phefs*13 |  |
|  | Hom | c.1141C>T | p.Gln381* | (Xu et al., 2016) |
|  | Com-het | c.2009_2010del | p.Glu670Glyfs*48 |  |
|  |  | c.633T>A | p.His211Gln |  |
|  | Com-het | c.1618G>A | p.Gly540Arg |  |
|  |  | c.970C>T | p.Gln324* |  |
|  | Hom | c.487T>C | p.C163R | (Xu et al., 2022) |
|  | Hom | c.1425G>A | p.W475* |  |
| Zygotic cleavage failure | hom | c.1369C>T | p.Arg457* | (Maddirevula et al., 2017) |
| Multilocus imprinting disturbances | Com-het | c.1114A>G | p.Thr372Ala | (Eggermann et al., 2021) |
|  |  | c.2069G>A | p.Trp690* |  |
|  | Com-het | c.1067G>A | p.Trp356Ter | (Cubellis et al., 2020) |
|  |  | c.1894C>G | p.Pro632Ala |  |
|  | Com-het | c.1429A>G | p.Met477Val |  |
|  |  | c.2080C>T | p.Pro694Ser |  |
|  | Het | c.2006delC | P.Thr669Lysfs*85 |  |
|  | Com-het | c.902G>A | p.Arg301Gln | (Begemann et al., 2018) |
|  |  | 1298C>T | p.Pro433Leu |  |
|  | Het | c.1046A>G | p.Asp349Gly |  |
|  | Het | c.[433A>G] | p.Lys145Glu |  |
| Recurrent hydatidiform moles | Hom | c.1796T>A | p.Ile599Asn | (Rezaei et al., 2021) |
|  | Com-het | c.2045G>A | p.Arg682Gln | (Qian et al., 2018) |
|  |  | c.1793A>G | p.Asn598Ser |  |

Hom, homozygous; Het, heterozygous; Com-het, compound-heterozygous.

**References:**

Begemann, M., Rezwan, F.I., Beygo, J., Docherty, L.E., Kolarova, J., Schroeder, C., Buiting, K., Chokkalingam, K., Degenhardt, F., Wakeling, E.L., Kleinle, S., González Fassrainer, D., Oehl-Jaschkowitz, B., Turner, C.L.S., Patalan, M., Gizewska, M., Binder, G., Bich Ngoc, C.T., Chi Dung, V., Mehta, S.G., Baynam, G., Hamilton-Shield, J.P., Aljareh, S., Lokulo-Sodipe, O., Horton, R., Siebert, R., Elbracht, M., Temple, I.K., Eggermann, T., Mackay, D.J.G. (2018). Maternal variants in NLRP and other maternal effect proteins are associated with multilocus imprinting disturbance in offspring. J MED GENET 55, 497-504.10.1136/jmedgenet-2017-105190.

Cubellis, M.V., Pignata, L., Verma, A., Sparago, A., Del Prete, R., Monticelli, M., Calzari, L., Antona, V., Melis, D., Tenconi, R., Russo, S., Cerrato, F., Riccio, A. (2020). Loss-of-function maternal-effect mutations of PADI6 are associated with familial and sporadic Beckwith-Wiedemann syndrome with multi-locus imprinting disturbance. CLIN EPIGENETICS 12.10.1186/s13148-020-00925-2.

Eggermann, T., Kadgien, G., Begemann, M., Elbracht, M. (2021). Biallelic PADI6 variants cause multilocus imprinting disturbances and miscarriages in the same family. EUR J HUM GENET 29, 575-580.10.1038/s41431-020-00762-0.

Liu, J., Tan, Z., He, J., Jin, T., Han, Y., Hu, L., Huang, S. (2021). Two novel mutations in PADI6 and TLE6 genes cause female infertility due to arrest in embryonic development. J ASSIST REPROD GEN.10.1007/s10815-021-02194-1.

Maddirevula, S., Coskun, S., Awartani, K., Alsaif, H., Abdulwahab, F.M., Alkuraya, F.S. (2017). The human knockout phenotype of PADI6 is female sterility caused by cleavage failure of their fertilized eggs. CLIN GENET 91, 344-345.10.1111/cge.12866.

Qian, J., Nguyen, N.M.P., Rezaei, M., Huang, B., Tao, Y., Zhang, X., Cheng, Q., Yang, H., Asangla, A., Majewski, J., Slim, R. (2018). Biallelic PADI6 variants linking infertility, miscarriages, and hydatidiform moles. EUR J HUM GENET 26, 1007-1013.10.1038/s41431-018-0141-3.

Rezaei, M., Suresh, B., Bereke, E., Hadipour, Z., Aguinaga, M., Qian, J., Bagga, R., Fardaei, M., Hemida, R., Jagadeesh, S., Majewski, J., Slim, R. (2021). Novel pathogenic variants in NLRP7, NLRP5, andPADI6 in patients with recurrent hydatidiform moles and reproductive failure. CLIN GENET 99, 823-828.10.1111/cge.13941.

Wang, X., Song, D., Mykytenko, D., Kuang, Y., Lv, Q., Li, B., Chen, B., Mao, X., Xu, Y., Zukin, V., Mazur, P., Mu, J., Yan, Z., Zhou, Z., Li, Q., Liu, S., Jin, L., He, L., Sang, Q., Sun, Z., Dong, X., Wang, L. (2018). Novel mutations in genes encoding subcortical maternal complex proteins may cause human embryonic developmental arrest. REPROD BIOMED ONLINE 36, 698-704.10.1016/j.rbmo.2018.03.009.

Xu, Y., Shi, Y., Fu, J., Yu, M., Feng, R., Sang, Q., Liang, B., Chen, B., Qu, R., Li, B., Yan, Z., Mao, X., Kuang, Y., Jin, L., He, L., Sun, X., Wang, L. (2016). Mutations in PADI6 Cause Female Infertility Characterized by Early Embryonic Arrest. The American Journal of Human Genetics 99, 744-752.10.1016/j.ajhg.2016.06.024.

Xu, Y., Wang, R., Pang, Z., Wei, Z., Sun, L., Li, S., Wang, G., Liu, Y., Zhou, Y., Ye, H., Jin, L., Xue, S. (2022). Novel Homozygous PADI6 Variants in Infertile Females with Early Embryonic Arrest. Frontiers in Cell and Developmental Biology 10.10.3389/fcell.2022.819667.

Zheng, W., Chen, L., Dai, J., Dai, C., Guo, J., Lu, C., Gong, F., Lu, G., Lin, G. (2020). New biallelic mutations in PADI6 cause recurrent preimplantation embryonic arrest characterized by direct cleavage. J ASSIST REPROD GEN 37, 205-212.10.1007/s10815-019-01606-7.

Zheng, W., Hu, H., Dai, J., Zhang, S., Gu, Y., Dai, C., Guo, J., Xu, X., Li, Y., Zhang, S., Hu, L., Gong, F., Lu, G., Lin, G. (2021). Expanding the genetic and phenotypic spectrum of the subcortical maternal complex genes in recurrent preimplantation embryonic arrest. CLIN GENET 99, 286-291.10.1111/cge.13858.
